# Bisphenol A in dairy products, amount, potential risks, and the various analytical methods, a systematic review

**DOI:** 10.1016/j.fochx.2024.101142

**Published:** 2024-01-17

**Authors:** Mohammad-Hossein Ghahremani, Mahmoud Ghazi-Khansari, Zahra Farsi, Najmeh Yazdanfar, Mahadi Jahanbakhsh, Parisa Sadighara

**Affiliations:** a Department of Toxicology & Pharmacology, School of Pharmacy, Tehran University of Medical Sciences, Tehran, Iran; b Department of Pharmacology, School of Medicine, Tehran University of Medical Sciences, Tehran, Iran; c Iranian Institute of R&D in Chemical Industries (IRDCI) (ACECR), Tehran, Iran; d Department of Environmental Health Engineering, School of Public Health, Iran University of Medical Sciences, Tehran, Iran

**Keywords:** Bisphenol A, Analytical method, Risk assessment, Dairy product, Milk

## Abstract

This systematic study deals with the amount of bisphenol A (BPA) in milk and dairy products, its analytical methods, and risk assessment. Milk is one of the drinks that has a high consumption. Bisphenol A can be present both in raw milk and its amount undergoes changes during the pasteurization process. This review was conducted by searching for the keywords Bisphenol A, BPA milk, dairy product, cheese, cream, butter, yogurt, measurement, detection, and analysis in different databases. The search was done in three databases, Scopus, PubMed and Science Direct. The largest number of studies on the determination of bisphenol A belonged to Asian and European countries. The amount of bisphenol A in milks was observed in the range from ND to 640 ng/mL. Furthermore, the amount of BPA in the tested cheese samples was observed in the ND range up to 6.1 ng/g and in the yogurt samples in the ND range up to 4.4 ng/g. The most used analytical method was based on liquid chromatography. The most used solvent for extraction was methanol or acetonitrile. HQ (Hazard Quotient) was also calculated in some studies. There was no risk in terms of milk consumption due to BPA contamination in extracted data.

## Highlights

• BPA was detected in dairy samples, but it was not dangerous in terms of risk assessment.
• One of the causes of BPA contamination in dairy products is their production processes.
• The most common method of measuring BPA is based on liquid chromatography.

## Introduction

Bisphenol A (BPA) is widely used. It is found in epoxy resins, polycarbonate, adhesives, electrical appliances, medical equipment, dental material flings, flame retardants, and food packaging (Cai et al., 2018, Cheng et al., 2017, Malone et al., 2010). 95 % of produced BPA is used in epoxy resins and polycarbonates (Huang et al., 2012). It is also used as additives in PVC(polyvinyl chloride) as a stabilizer (Grumetto et al., 2013). The environmental pollution of Bisphenol A is high due to its widespread use. BPA has been identified in urban sewage, water surface, water sediments, air and soil (Santonicola, Ferrante, Leo, Murru, Anastasio, & Mercogliano, 2018). In a study, the amount of BPA in the leachate of sewage waste was measured in the range between 1.3 and 17,200 μg/L (Yamamoto, Yasuhara, Shiraishi, & Nakasugi, 2001). This compound has even been detected in house dust (Pisciottano et al., 2022).

Due to its wide use, the compound can enter the environment and later food. Also, due to the use of epoxy resin and polycarbonate in food packaging, there is a possibility of contamination of food with this compound. It is estimated that 3 % of polycarbonates and 10 % of epoxy resins produced come into contact with food (Liao & Kannan, 2014). The amount of BPA migration from packaging to food contents inside them has been approved in the European Union at 3 mg/kg and Japan at 2.5 mg/kg (Kang & Kondo, 2003). Currently, the European Union has reduced this amount to 0.5 mg/kg (Mercogliano, Santonicola, Albrizio, & Ferrante, 2021). The amount of tolerable daily intake is estimated at 4 μg/kg bw per day (Yao et al., 2020).

BPA an endocrine disruptor (Cheng et al., 2017). Regarding compounds that interfere with endocrine glands, bisphenol A has the most attention and also the most danger (Herrero, Quintanilla-López, Fernández, & Gómara, 2021). This compound binds to estrogen receptors (Cai et al., 2018, Sadighara et al., 2022, Sadighara and Ghanati, 2022). This compound can synergize with other xenoestrogens in the body and have adverse health effects (Grumetto et al., 2013). It also leads to early puberty. Due to having a weak estrogenic property, it leads to pituitary FSH and LH secretion (Konieczna, Rutkowska, & Rachon, 2015). Studies have shown that mothers who use milk containing BPA during pregnancy, their babies will experience changes in the level of adiponectin and leptin (Minatoya et al., 2018). Bisphenol A suppresses the release of adenopectin from adipose tissue. Adenopectin increases insulin sensitivity. Therefore, when it is suppressed, we will see an increase in insulin resistance (Preethi et al., 2014). Furthermore, bisphenol A decreases aromatase enzyme and decreases testosterone synthesis (Preethi et al., 2014). This combination also leads to obesity, diabetes and infertility in men (Liu, Li, Sun, Yang, Zheng, & Wang, 2019). Dermal and inhalation exposure has been reported for bisphenol A (Mercogliano et al., 2021). But, the most important exposure to BPA is oral (Herrero et al., 2021, Maragou et al., 2006, Rodríguez-Gómez et al., 2014). BPA does not accumulate in the body. It is metabolized in the liver and conjugated with sulfate or glucuronic acid and excreted from the body with urine. Non-conjugated BPA is found in milk, and its conjugated form does not enter milk due to the presence of fat in milk (Kishikawa & Kuroda, 2009).

Milk is a relatively cheap food and rich in nutrients such as vitamins, protein, saturated fat and minerals (Mesa, Kabir, Samanidou, & Furton, 2019). Global milk consumption has increased by 20 % compared to the last ten years (Calahorrano-Moreno, Ordoñez-Bailon, Baquerizo-Crespo, Dueñas-Rivadeneira, Montenegro, & Rodríguez-Díaz, 2022). This nutritious composition can contain some environmental contaminants such as heavy metals, pesticides, drug residues and some chemicals from industrial activities. Some resins contain bisphenol, which is used in milk containers (Malone et al., 2010). Some studies indicate that the most contamination of milk with BPA is through their storage tanks (Santonicola, Albrizio, Ferrante, & Raffaelina, 2021).

The amount of BPA in food is usually low, but it should be noted that its chronic exposure can be a serious health risk. Therefore, in high-consumption food products, its amount should be measured regularly. A comprehensive review of BPA in canned foods has been conducted (Cai et al., 2018). However, fewer studies have been done on dairy products. This systematic study for the first time deals with the amount and method of measuring bisphenol A in dairy products. The results of risk assessment were also discussed.

## Method

### Search strategy

The keywords of this systematic review were searched in databases on August 10, 2023. The search formula is as follows: (bisphenol A or BPA) and (milk or “dairy products” or cheese or cream or butter or yogurt) and (measurement or detection or analysis). The search was done with keywords determined by two authors independently (Z.F and N.Y). The search results of both authors were the same.

### Inclusion and exclusion criteria

The inclusion criteria of this study include manuscripts that measure the amount of BPA in milk and dairy products. The studies that were based on the developed method were excluded from this systematic study. Furthermore, review studies, non-English, conference and book chapters were also excluded. Furthermore, in the initial screen of articles, a number of manuscripts were excluded from this systematic study due to animal studies, food other than milk and dairy product, bioassays, and toxicity evaluation.

## Result

### Search procedure

**Fig. 1:**
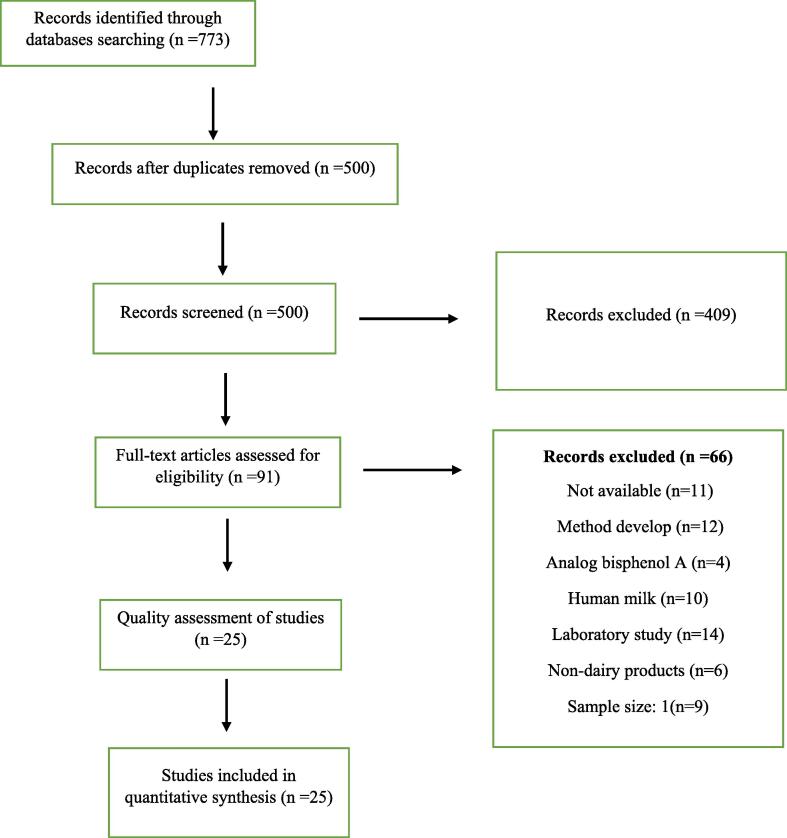
The diagram of study.

The search of this study was done with the keywords approved by the author members in PubMed, Science Direct and Scopus databases. 773 manuscripts were entered in the Endnote software as a result of the search (Fig. 1). Duplicate manuscripts were removed with the option available in the software. The number of manuscripts reached 500 after removing duplicate manuscripts. The next step was to screen the manuscripts. The title and abstract of the article were read. Articles that met the inclusion criteria were included in this systematic review. 91 manuscripts were selected for full text review. 5 factors were considered for the qualitative evaluation of the manuscripts. These factors include the sample size, valid measurement method, presenting the details of the sample preparation method accurately, considering the confounding factors and using accurate statistical methods and presenting the results transparently.

### Data extraction

After screening and quality assessment, 25 manuscripts were selected for data extraction. The extracted data included the name of the author, year of publication, country, analytical method, a summary of the sample preparation, risk assessment results, and the amount of bisphenol A.

### Geographical distribution of studies

**Fig. 2:**
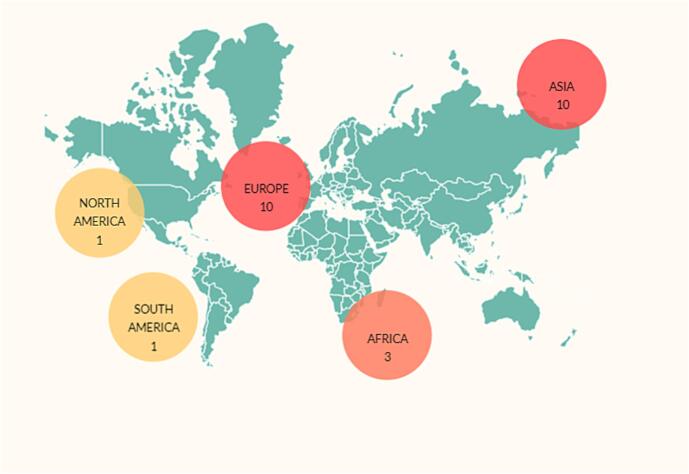
Geographical distribution of selected manuscripts, Africa: 3, Asia: 10, Europe: 10 and, North America: 1, South America: 1.

Fig. 2 shows the geographical distribution of manuscripts selected for data extraction. Most studies have been done in Asia and Europe. Among the Asian countries, China had the largest share. This Fig confirms the need to conduct more extensive studies in other continents and countries.

## Discussion

Milk goes through several stages from the production stage to the stage when it reaches the consumer (Mercogliano et al., 2021). The possibility of contamination in milk is either through the environment or the factory during their processing. BPA is released into the environment following industrial activities and pollutes underground water and soil (Konieczna et al., 2015). Milk from animals living in contaminated areas is likely to be contaminated with BPA (Konieczna et al., 2015). During pasteurization and processing, milk and dairy products are in contact with many equipment, such as milking equipment, milk storage tanks, milk transport pipes. In this systematic study, the reported amount of bisphenol A in dairy products, its measurement method and risk assessment are discussed.

### Reported amounts of BPA in dairy products

The amount of BPA in milk samples were almost in the same range. High amounts were observed in only two studies. One study in Italy with an amount of 521.0 ng/mL and another in China with an average amount of 0.64 μg/mL (640 ng/mL) are significant in the data extracted (Table 1). Both of these studies warn about contamination of dairy products with high amounts of BPA.

**Table 1:** The extracted data according to the protocol.

Type of dairy product Amount/sample size	Analytical method	Sample preparation	HQ/EDI	Country	Author/year
Milk: 127.2 ± 2.8 µg/kg Yoghurt: ND N = 3	UHPLC-MS/MS	QuEChERS procedure	–	China	Cheng/2017
Milk ND- 521.0 ng/mL N = 69	LC-MS/MS	Extraction: mixing 10 ml milk with 10.0 ml of methanol, sonication in ultrasonic apparatus Clean up: SPE	–	Italy	Grumetto/2013
Milk 0.472 to 1.014 mg/dm3 N = 69	HPLC–UV	Extraction: mixing milk with 20 ml of (methanol: water), adjusted pH to 3(with HCL) Clean up: SPE	–	Iran	Hadjmohammadi/2010
Milk NQ-59 ng/g N = 42	UHPLC-QqQ	Using a matrix solid-phase dispersion	–	Spain	Herrero/2021
Non canned dairy products < 3 canned dairy products range:21–43 µg/kg	HPLC–FLD	Double extraction with *n*-hexane and acetonitrile Clean up: SPE	–	Japan	Kang/2003
Milk range: ND–2.6 ng/ ml N = 15	HPLC–FLD	Deproteinization with TCA, extraction with methanolClean up:Solid-phase microextraction (SPME)	–	China	Liu/2008
Milk 0.64 ± 0.02 μg/mL N = 3	HPLC–UV	Derivatization of samples with p-methylaniline	–	China	Liu/2018
Caned milk Ranged:<1.7–15.2 ng/g N = 9	LC-ESI-MS	Extraction is done with a combination of water and methanol Clean up: SPE	–	Greece	Maragou2006
Mean Raw milk: 0.265 Milk after pasteurization: 0.164 Milk after packaging: 0.016 µg/L N = 28	HPLC–FLD	Extraction: mixing 1 ml milk with 3 ml of water, sonication in ultrasonic apparatus Clean up: SPE	EDI 1–3: 0.0131 16–65: 0.0009 (µg/kg of BW per day)	Italy	Mercogliano/2021
Milk Range:9.6–23.5 ng /mL N = 10	HPLC–UV	Deproteinization with TCA(15 ml milk with 200 mg TCA), extraction with chloroform and acetonitrile	–	Iran	Rostamzadeh/2021
Raw buffalo milk(N = 46): 0.5–5.6Packaged cow's milk (N = 15):ND-2.8 ng/mL	UHPLC–MS/MS	–	EDI: 0.002391665 µg/kg bw/day	Italy	Pisciottano/2020
Raw milk ND- 2.34 µg/L N = 144	HPLC–FLD	Extraction: mixing 2.5 ml milk with 7.5 ml of water, sonication in ultrasonic apparatus Clean up: SPE	HQ < 1	Italy	Santonicola/2018
Mean Raw milk: 0.580 Milk from milk cooling tank 0.797 µg/L N = 72	HPLC–FLD	Extraction: mixing 2.5 ml milk with 7.5 ml of water, sonication in ultrasonic apparatus Clean up: SPE	HQ < 1	Italy	Santonicola/2018
Commercial Milk ND- 0.49 µg/kg N = 10	LC–ESI–MS/MS	Extraction: matrix solid phase dispersion Clean up: SPE	HQ < 1	China	Shao/2007
Commercial Milk ND–10.8 µg/kg N = 17	HPLC-MS/MS	Extraction: mixing 3 g milk with 6 ml of acetonitrile, evaporating of supernatant, adding formic acid (0.2 %) Clean up: using cartilage	–	China	Liao/2015
Commercial Milk 0.09––0.26 µg/kg N = 17	UPLC-MS/MS	Extraction: mixing milk with acetonitrile, sonication in ultrasonic apparatus, then centrifuge, dryness and Reconstitution with MeOH/PBS Clean up: IAC	–	China	Yao/2018
Commercial Milk HDPE bottles Range: 1.17–1.29 µg/kg N = 2	GC–MS	Extraction: mixing milk with methanol, sonication in ultrasonic apparatus, Clean up: C-18 cartridges	–	Spain	Casajuana/2004
Milk Range: 0.12 and 0.36 ng/g N = 51	UHPLC-MS/MS	QuEChERS procedure	–	Brazil	Souza/2023
Cheese <0.002 mg/kg N = 3	UPLC-MS/MS	Mixing cheese with acetonitrile, adding Carrez solution I and II to the supernatant, then centrifuging and injecting the supernatant into the device	–	Tunisia	Beltifa/2018
Cheese Range: 2.06 to 2.84 µg/kg	HPLC-MS/MS	QuEChERS procedure	HQ < 1	Italy	Liotta/2022
Butter(N = 2), and yoghurt(N = 3) All sample were < 0.002 mg/kg	UPLC-MS/MS	Mixing samples with acetonitrile, adding Carrez solution I and II to the supernatant, then centrifuging and injecting the supernatant into the device	–	Tunisia	Beltifa/2017
Milk9.4 (±4.2) µg/kg N = 27	LC-LTQ/Orbitrap MS	QuEChERS procedure	HQ < 1	Greece	Boti/ 2021
Mean Cottage cheese: 6.1 ng/g yoghurt:3.3 ng/g packaged milk:2.5 ng/g	GC–MS	It was extracted three times with *n*-hexane. After centrifugation, the hexane layer was dried with nitrogen.	–	India	Chakraborty/2022
Skimmed cow’s milk: 3.4 ± 0.2 Semi-skimmed cow’s milk: 3.6 ± 0.2 Whole cow’s milk: 4.6 ± 0.3 Whole sheep’s milk: 2.5 ± 0.2 whole goat’s milk: 0.98 ± 0.06 Yoghourt cow’s: 0.99 ± 0.06 Yoghourt goat́s: 4.4 ± 0.3 µg/kg N = 3 for each group	GC–MS	Extraction: mixing samples with acidified ACN Clean up: SPE	–	Morocco	Colón/2021
Butter ND N = 4	LC-QTOF-MS	Extraction: mixing samples with methanol, sonication in ultrasonic apparatus,	–	Canada	Tian/2022

Note: ND; not detected. UHPLC-QqQ; ultra-high performance liquid chromatography coupled to triple quadrupole mass spectromete, TCA; trichloroacetic acid, IAC; immunoaffinity column, HDPE bottles; high-density polyethylene, EDI; Estimated daily intake.

In the Mercogliano/2021 study, the amount of BPA in milk was measured during the production process until the consumption of milk. In this study, it was measured in three stages: raw milk, milk after pasteurization and milk after packing (Mercogliano et al., 2021). Contamination in raw milk is caused by the environment and animal diet (Mercogliano et al., 2021). In this study, the amount of BPA in packaged milk was lower than raw milk (Mercogliano et al., 2021). It is probably caused by the reduction of milk fat. BPA is a lipophilic compound and its amount decreases with the reduction of fat. Another cause that probably plays a role in this is the equipment used in the milk collected from the bisphenol-free factory. Contrary to the results of this study, we can refer to the results of the Santonicola/2018 study. In this study, the amount of BPA in milk sampled from cooling tank was more than in raw milk. Usually, milk samples are stored in this tank for 2 to 3 days. Therefore, there is a possibility of BPA migration from the tanks to the contents of the milk (Santonicola et al., 2018). In this study, milk was stored in steel tanks with rubber sealants.

In the Grumetto/2013 study, the amount reported in positive samples was low. However, milk is one of the food items with high per capita consumption. Small amounts of BPA will be problematic during chronic exposure. Also, the authors stated that the amount of bisphenol was not affected by the type of packaging. The impact of the production process was blamed mostly on the amount of bisphenol (Grumetto et al., 2013).

In the study of Herrero/2021, bisphenol A was detected in 88 % of milk samples (Herrero et al., 2021). In this study, the amount of BPA in milk with different types of packaging was investigated. The highest amount of BPA in milk was observed in HDPE (high-density polyethylene) bottles and then in metal pail packaging. Metal pails have an epoxy coating, so it is normal to increase the amount of bisphenol in them, but the amount of bisphenol in milk with HDPE packaging is probably due to the production process (Herrero et al., 2021). In the study of Kang/2003, a significant difference was observed between the amount of BPA in canned and non-canned dairy products (Table 1). This is caused by the migration of BPA from the epoxy coatings in the cans when the product is heated during the process (Kang and Kondo, 2003). In the study of Liu/2008, the range of BPA in the tested milk was ND-2.6 ng/mL. The amount of BPA was not observed in milk packed with plastic bottles and tetra packs. But, it was seen in HDPE (high-density polyethylene) packaging. The polyethylene used in these packages was food grade and cannot be a source of BPA. Probably, this packaging was contaminated with BPA during processing (Liu, Ji, Zhang, & Liu, 2008).

In the study by Beltifa et al., the amount of BPA in cheeses was lower than LOQ (Beltifa et al., 2018). Contrary to these results, in the Chakraborty/2022 study from India, the amount of BPA in cottage cheese was higher than other dairy products (Chakraborty et al., 2022). The authors of the recent research know that the reason for the high level of BPA in cheeses is due to the high fat content of this product, which has caused the accumulation of BPA in them (Chakraborty et al., 2022). In the study of Liotta/2022, the amount of bisphenol in cheeses was reported in the range of 2.06 to 2.84 µg/kg. The researchers of this research declare this amount due to the introduction of bisphenol during the production process and packaging materials in cheeses (Liotta et al., 2022).

In the Colón/2021 study, the amount of bisphenol in cow, sheep and goat milk was measured. The amount of bisphenol in cow's milk was higher than the other two species (Colón, Rascón, Hejji, Azzouz, & Ballesteros, 2021). On the contrary, goat's milk yogurts were more than cow's milk yogurts. Of course, it is worth mentioning that factors such as production process equipment, type of packaging and environmental pollution can play a role (Colón et al., 2021).

There were few studies about butter, and the amount of bisphenol in all of them was reported as ND (Beltifa et al., 2018, Tian et al., 2022).

### Analytical method for measuring BPA in dairy products

Among different solvents, acetonitrile is the most widely used for extracting BPA from solid food (Ballesteros-Gómez, Rubio, & Pérez-Bendito, 2009). A high percentage of recoveries has been obtained due to the use of acetonitrile solvent in the extraction phase of BPA in food (Talari, Ganji, & Tiruveedula, 2023). In the preparation of the samples in the reviewed manuscripts, QuEChERS method was one of the observed methods. In Cheng/2017 study, QuEChERS method was used for sample preparation. Acetonitrile and hexane were used in this study. Then MgSO4 and NaCl were added. The solution was centrifuged, the hexane layer was discarded and the acetonitrile layer was kept. This method has advantages due to the need for fewer solvent and time (Xiong et al.., 2018). Also, the remaining water is removed in this method (Souza, Krauss, Sartori, & Abrantes, 2023). Furthermore, in the studies of Souza/2023 and Boti/ 2021, this method was used to prepare milk samples (Boti et al., 2021, Souza et al., 2023). In this method, acidified acetonitrile, MgSO4 and NaCl were used for the extraction step. In the clean-up stage, MgSO4 was used along with C18 and PSA. GCB (graphitized carbon black) was also used in the optimization process. But due to the reduction of the recovery percentage, it was not used in the preparation of real samples (Boti et al., 2021). It has also been observed in experimental studies that the peaks of bisphenol in acidic solutions are higher than in basic solutions (Lv et al., 2014). Therefore, in the first stage of sample preparation, acidifying acetonitrile can be useful in this regard.

In the study of Liu/2018, in the preparation of the samples, derivatization was done with p-methylaniline compound, and the percentage of recovery was reported to be 96.3 % (Liu et al., 2019). In this way, stronger responses are recorded in the UV spectrum (Liu et al., 2019). Also, in this study and the study of Rostamzadeh/2021, deproteinization was done with TCA. The best TCA concentration is reported to be 200 mg for 15 ml of milk (Rostamzadeh, Nemati, Farajzadehd, & Mogaddam, 2021). TCA is usually used for protein precipitation and analyte separation from the matrix in sample preparation. Of course, it is worth mentioning that its amount needs to be optimized (Yan, Zhou, Zhu, & Chen, 2009).

In only one study in selected manuscripts, an immunoaffinity column was used in the preparation of real milk samples. These columns are based on monoclonal antibodies. The authors of the manuscript recommend this method for complex matrices (Yao et al., 2018). The clean-up step is a critical step in the isolation of compounds that are present in small amounts in food. These columns have trapped BPA antibodies. It has been reported that the recovery percentage can be up to 98 % (Braunrath, Podlipna, Padlesak, & Cichna-Markl, 2005). These columns are stable and can be stored for at least three months, and they are highly sensitive even for the separation of bisphenol in biological samples (Zhao, Liu, Li, Zhang, & Chang, 2003). These columns are suitable for removing interfering matrix factors and BPA enrichment (Cichna-Markl, 2012). Also, Carrez reagent was used in sample preparation only in two studies. These study were conducted on cheese, milk, butter, and yoghurt samples in Tunisian markets (Beltifa et al., 2018, Beltifa et al., 2017). Carrez reagent is used for precipitation of proteins and carbohydrates (Lestido-Cardama, Sánchez, Sendón, Rodríguez-Bernaldo de Quirós, & Barbosa-Pereira, 2022).

Only in the Casajuana/2004, Colon/2021, and Chakraborty/2022studies, BPA was measured with GC–MS (Casajuana and Lacorte, 2004, Chakraborty et al., 2022, Colón et al., 2021). Using the GC–MS method to measure bisphenol A in food matrices requires derivatization. Phenolic compounds need derivatization by measuring with GC–MS. Derivatization increases the sensitivity of the measurement method and avoids reporting false positives (Colón et al., 2021). In the Colón/2021 study, derivatization was done with N,O-bis(trimethylsilyl)trifluoroacetamide and trimethylchlorosilane (Colón et al., 2021). Bromoacetonitrile (BAN) is also used for derivatization (Szyrwińska, Kołodziejczak, Rykowska, Wasiak, & Lulek, 2007). Another the most effective substances for derivatization is heptafluorobutyric anhydride (Cai et al., 2018). Derivation makes it give sharper peaks. However, the process of derivatization is time-consuming (Kawaguchi et al., 2006).

Determination of bisphenol A in most studies was based on liquid chromatography. The detector used is the fluorescence or MS detector. Only in three studies, HPLC–UV was used. It has been announced in the results that the percentage of recovery is high (Hadjmohammadi & Saeidi, 2010). In the study of Maragou 2006, LC-ESI-MS was used to measure BPA and C18 cartridges were used to prepare canned milk samples. The recovery rate was 97–104 % (Maragou et al., 2006). In a study, the difference between LC/MS/MS and HPLC/FLD methods in the measurement of BPA was investigated. There was a difference between these two methods. The authors found that the difference was measured more by the LC/MS/MS method in samples containing less BPA and measured less than the actual amount in samples containing BPA by HPLC/FLD (Yi, Kim, & Yang, 2010).

### Risk assessment of BPA in dairy products

So far, the permissible limit for the amount of BPA in milk and dairy products has not been determined. Therefore, one of the safety evaluations of these products in terms of BPA is to determine HQ and EDI. HQ (Hazard Quotient) is to consider non-carcinogenic risks (Sadighara and Ghanati, 2022, Sadighara et al., 2022). The EDI in Mercogliano/2021 study was calculated for different age groups, which was higher in children than in adults (Mercogliano et al., 2021). In this study, the calculated amount of EDI for infants and children is lower than the TDI compiled for BPA. However, due to the harmful effects of BPA, the authors recommend specific TDI for infants (Mercogliano et al., 2021). Exposure of children to bisphenol-containing milk has serious health risks for them (Otaka, Yasuhara, & Morita, 2003). Also, the amount of exposure in the Pisciottano/2020 study was conducted in Italy, and the calculated value was lower than the TDI (Pisciottano et al., 2022). Similar results were observed in the Santonicola/2018 study and the HQ was less than one (Santonicola et al., 2018). In the Shao/2007 study in China, BPA was measured in 10 milk samples using the LC–ESI–MS/MS method. BPA was detected only in one sample and it was found that there is no risk for consumers in this regard through the risk assessment (Shao, Han, Tu, & Huang, 2007).

## Conclusion and future research

This systematic review summarized the amount of BPA in milk and dairy products, its extraction and sample preparation, analytical methods and risk assessment. The risk assessment reported so far has shown that milk is safe because of BPA. Most of the studies were done on milk. Limited studies were also done on cheese, yogurt and butter. The most analytical methods for measuring BPA in food were based on liquid chromatography. These methods are well developed. Analytical methods for measuring BPA in 12 % of the studies were based on gas chromatography. Gas chromatography-based methods require derivatization. In most of the studies, there was no correlation between the type of packaging and the amount of BPA in dairy products. The possibility of this is due to the fact that this combination is not usually used in the packaging of dairy products. Some studies were about BPA analogs. It is recommended to carry out a systematic review in this regard and summarize the amount reported in food items.

## CRediT authorship contribution statement

**Mohammad-Hossein Ghahremani:** . **Mahmoud Ghazi-Khansari:** Funding acquisition. **Zahra Farsi:** Writing – original draft. **Najmeh Yazdanfar:** Validation. **Mahadi Jahanbakhsh:** Writing – original draft. **Parisa Sadighara:** Writing – review & editing.

## Declaration of competing interest

The authors declare that they have no known competing financial interests or personal relationships that could have appeared to influence the work reported in this paper.

## Data Availability

The data that has been used is confidential.
